# Survival Impact of Neoadjuvant Chemotherapy With Bevacizumab Followed by Radical Hysterectomy for Locally Advanced Cervical Cancer With Lymph Node Metastasis: Insights From a Single-Institution Experience

**DOI:** 10.7759/cureus.86032

**Published:** 2025-06-15

**Authors:** Takeshi Motohara, Akiho Nishimura, Munekage Yamaguchi, Hidetaka Katabuchi, Eiji Kondoh

**Affiliations:** 1 Department of Obstetrics and Gynecology, Kumamoto University, Kumamoto, JPN

**Keywords:** bevacizumab, cervical cancer, lymph node metastasis, neoadjuvant chemotherapy, radical hysterectomy

## Abstract

Objective: To evaluate the therapeutic efficacy of neoadjuvant chemotherapy (NAC) with bevacizumab prior to radical hysterectomy in locally advanced cervical cancer with lymph node metastasis.

Methods: This single-institution, retrospective study was conducted at Kumamoto University Hospital (Kumamoto, JPN). The medical records of six consecutive patients with stage IIIC cervical cancer who received NAC with bevacizumab followed by radical hysterectomy were reviewed. Patients were treated between January 2022 and January 2024, with follow-up continuing through April 2025. Treatment responses, survival outcomes, and perioperative outcomes, including surgical complications and adverse events, were analyzed.

Results: All six patients had tumors ≥4 cm with parametrial invasion (T2b); two had stage IIIC1r and four had IIIC2r disease. All patients completed three to four cycles of platinum-based NAC with bevacizumab and subsequently underwent radical surgery. Substantial tumor regression after NAC was observed in all cases, and notably, pathological complete response was achieved in three patients (50.0%). At a median follow-up of 25.5 months, five patients (83.3%) remained recurrence-free, and no deaths had occurred. One patient with pelvic nodal recurrence underwent complete resection followed by concurrent chemoradiotherapy, achieving sustained disease-free status. All surgeries were completed without severe perioperative complications. No severe bevacizumab-related adverse events were observed.

Conclusion: The NAC with bevacizumab followed by radical hysterectomy was feasible, well tolerated, and demonstrated promising efficacy in patients with locally advanced cervical cancer accompanied by lymph node metastasis. This strategy demonstrated remarkably high clinical response and survival outcomes, along with favorable perioperative safety, supporting its potential as a multimodal treatment option for selected high-risk patients.

## Introduction

Cervical cancer remains a significant global health challenge and continues to be a leading cause of cancer-related mortality in women, particularly in developing countries [1,2]. Despite advances in screening programs and the widespread implementation of human papillomavirus (HPV) vaccination, many cases are still diagnosed at locally advanced or metastatic stages, where treatment options remain complex and outcomes continue to be suboptimal [3,4].

The management of cervical cancer is primarily determined by disease stage at diagnosis. For early-stage disease (International Federation of Gynecology and Obstetrics (FIGO) stages I-IIA), treatment typically involves radical hysterectomy with pelvic lymphadenectomy or definitive radiotherapy, often combined with concurrent chemotherapy. In contrast, for locally advanced cervical cancer (FIGO stages IIB-IVA), concurrent chemoradiotherapy (CCRT) with platinum-based regimens is the standard of care, having demonstrated survival benefits. For recurrent or distant metastatic disease, systemic chemotherapy, targeted therapies, and immune checkpoint inhibitors have increasingly been incorporated into treatment strategies, reflecting advances in precision oncology [3,5,6].

Neoadjuvant chemotherapy (NAC) followed by radical hysterectomy has been proposed as an alternative approach for patients with locally advanced cervical cancer [7-11]. The rationale for NAC lies in tumor downstaging, which may improve the likelihood of complete surgical resection, preserve postoperative urinary function, and potentially reduce the need for adjuvant radiotherapy, minimizing long-term morbidity [3,7]. Multiple studies have shown that platinum-based NAC can achieve objective response rates ranging from 69.4% to 90.2% in locally advanced cervical cancer [12,13]. However, despite these encouraging outcomes, NAC followed by radical hysterectomy has not yet been established as the standard treatment, largely due to heterogeneity in clinical outcomes and the recognized efficacy of CCRT [11,14]. In clinical practice, however, CCRT presents several clinical challenges, as approximately 30% of patients experience disease recurrence following therapy and may develop severe late-onset toxicities, including radiation-induced lymphedema, cystitis, proctitis, pelvic fractures, and vesicovaginal or rectovaginal fistulas [15].

Preliminary reports have explored the addition of bevacizumab, an anti-vascular endothelial growth factor (VEGF) monoclonal antibody, to NAC regimens to enhance tumor response through angiogenesis inhibition and improve oncological outcomes [16-18]. Bevacizumab has demonstrated significant survival benefits in metastatic and recurrent cervical cancer when combined with systemic chemotherapy [19,20], leading to growing interest in its potential utility in the neoadjuvant setting for locally advanced disease. Nevertheless, its clinical efficacy, safety profile, and perioperative feasibility in this neoadjuvant treatment context remain poorly defined, underscoring the need for further research. Therefore, in this retrospective study, we aimed to evaluate the therapeutic impact of NAC with bevacizumab prior to radical hysterectomy in patients with locally advanced cervical cancer accompanied by lymph node metastasis, focusing on oncological efficacy and perioperative safety in a real-world setting.

## Materials and methods

Study design and setting

This single-center, retrospective study was conducted at Kumamoto University Hospital to evaluate the oncological and perioperative outcomes of patients diagnosed with FIGO 2018 stage IIIC cervical cancer accompanied by lymph node metastasis who received NAC with bevacizumab followed by radical hysterectomy. The medical records of six consecutive patients treated between January 2022 and January 2024 were reviewed, with follow-up conducted until April 2025. All excised tissues were histologically evaluated by experienced pathologists per the 2020 WHO classification [21]. Staging was performed following the FIGO 2018 staging system for cervical cancer [3,22,23]. This study was approved by the Ethics Committee for Epidemiological and General Research at the Faculty of Life Science, Kumamoto University (approval no. 3235), and written informed consent was obtained from all patients in compliance with institutional guidelines.

Treatment protocol

All patients received an NAC regimen consisting of platinum-based chemotherapy in combination with bevacizumab. Following NAC, patients underwent radical hysterectomy with pelvic lymphadenectomy for FIGO stage IIIC1r disease and additional para-aortic lymphadenectomy for FIGO stage IIIC2r disease, performed by experienced gynecologic oncologists following standardized institutional protocols. Chemotherapy agents, dosing schedules, and surgical procedures were determined by a multidisciplinary tumor board based on individual patient characteristics. Of note, nerve-sparing radical hysterectomy was performed in all patients according to our institutional protocol based on the Okabayashi approach [24], in which the paravaginal space between the posterior leaf of the vesico-uterine ligament and the vaginal blood vessels is developed to allow selective preservation of the bladder branch of the inferior hypogastric plexus while dividing the uterine branch.

Regarding the treatment course, patients received either three or four cycles of NAC prior to radical surgery. The standard NAC regimen consisted of paclitaxel (175 mg/m²), carboplatin (dosed to an area under the curve of 5), and bevacizumab (15 mg/kg), administered once every three weeks. One patient who developed an allergic reaction to paclitaxel was switched to an alternative NAC regimen consisting of nedaplatin (80 mg/m²) and irinotecan (CPT-11; 60 mg/m²). Clinical response to NAC was assessed after the second or third cycle using pelvic examination and MRI to determine the optimal timing for radical surgery. All patients underwent radical surgery three to four weeks after the final chemotherapy cycle, with bevacizumab omitted in the last cycle to reduce perioperative complications and adverse events. Patients who underwent radical surgery received adjuvant therapy, based on the therapeutic efficacy of NAC, as assessed by the pathological findings of the resected specimen, and histological type, as well as protocol-defined criteria and established clinical guidelines.

Follow-up and outcome assessment

Postoperative follow-up was conducted through routine clinical evaluations, including physical and pelvic examinations, laboratory assessments, and imaging studies, until April 2025. The primary outcome was oncological efficacy, assessed by recurrence-free survival and overall survival. Secondary outcomes included perioperative safety and treatment-related adverse events.

## Results

Patient characteristics

**Figure 1:**
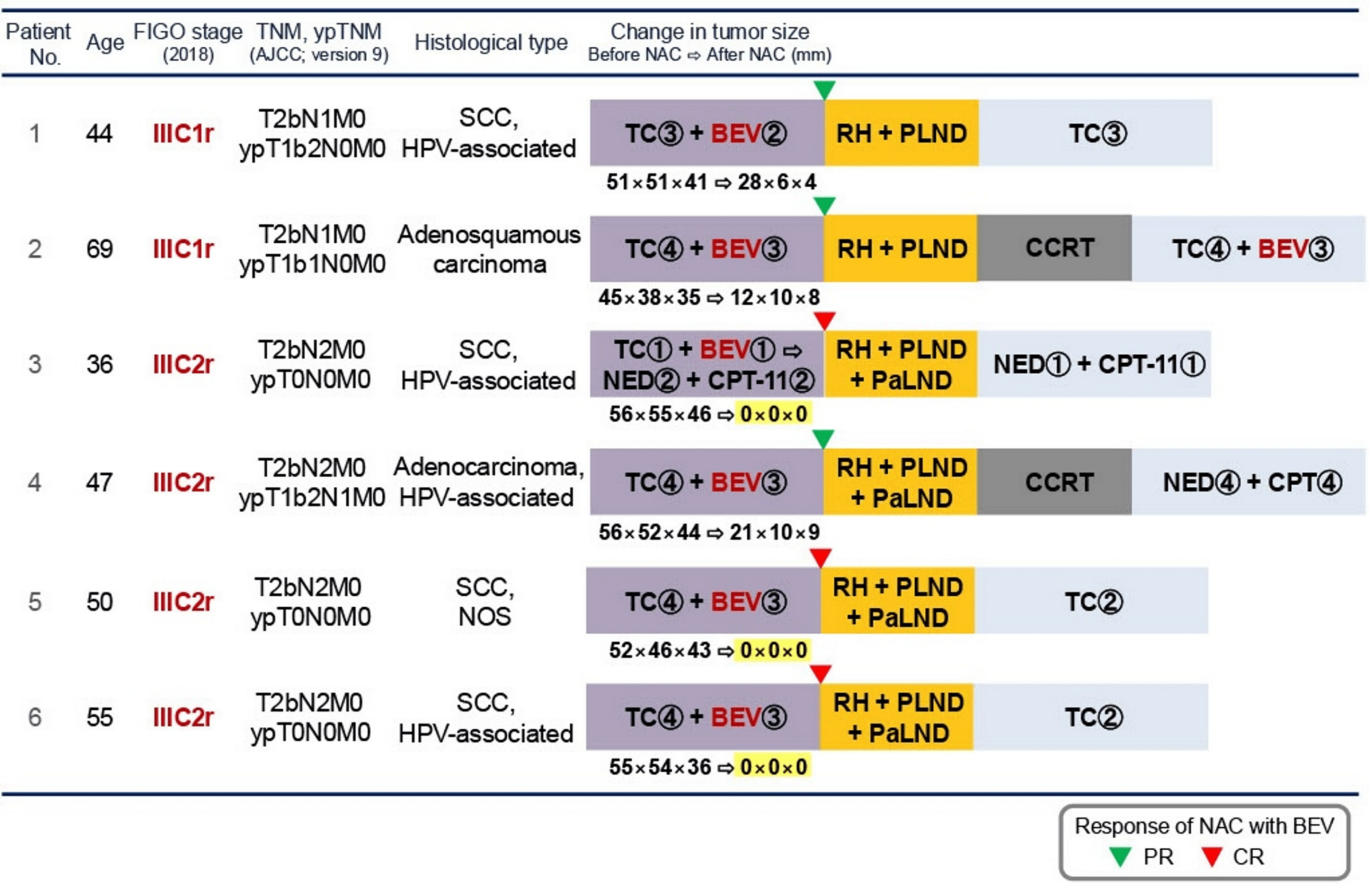
Clinical characteristics, treatment courses, and tumor responses of six patients with FIGO 2018 stage IIIC1r or IIIC2r cervical cancer who received NAC with bevacizumab followed by radical hysterectomy Tumor size measurements before and after NAC are presented for each patient. NAC: Neoadjuvant chemotherapy; FIGO: International Federation of Gynecology and Obstetrics; TNM: Tumor, node, metastasis; ypTNM: Post-neoadjuvant therapy TNM; AJCC: American Joint Committee on Cancer;  SCC: Squamous cell carcinoma; TC: Paclitaxel and carboplatin; BEV: Bevacizumab; NED: Nedaplatin; CPT-11: Irinotecan; RH: radical hysterectomy; PLND: Pelvic lymphadenectomy; PaLND: Para-aortic lymphadenectomy; CCRT: Concurrent chemoradiotherapy; HPV: Human papillomavirus; NOS: Not otherwise specified; PR: Partial response; CR: Complete response

A total of six patients with FIGO stage IIIC cervical cancer were included in this study. The clinicopathological characteristics of the patients, along with details of their treatment courses, are summarized in Figure 1. The median age of the study was 48.5 years (range: 36 to 69 years). Histopathological examination revealed that four patients had squamous cell carcinoma, whereas adenosquamous carcinoma and adenocarcinoma were each observed in one patient (Figure 1).

**Figure 2:**
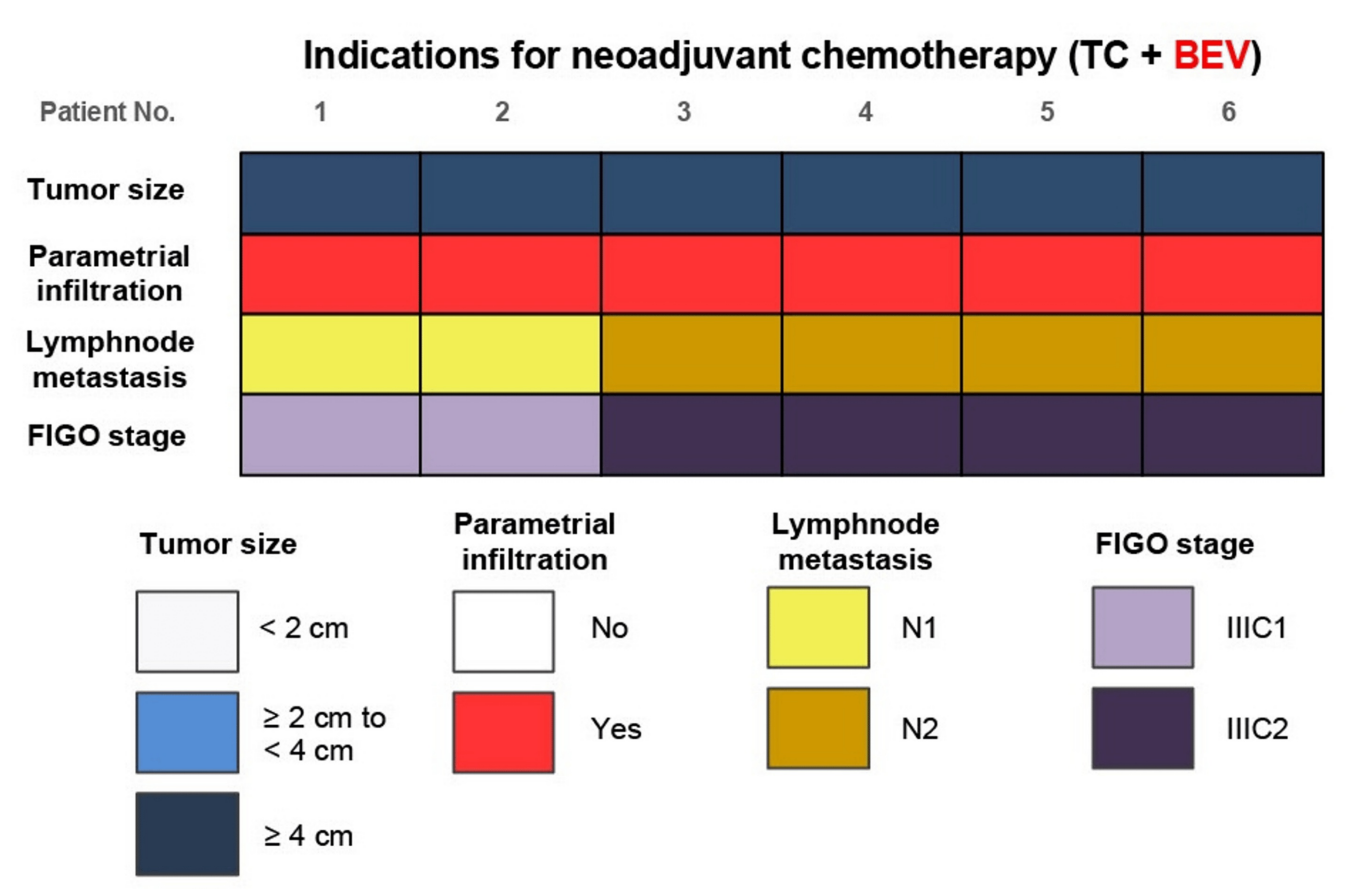
Pre-treatment staging and assessment of parametrium invasion and lymph node involvement in patients with locally advanced cervical cancer, based on pelvic examination and imaging studies. Two patients were diagnosed with FIGO stage IIIC1r, indicating pelvic lymph node metastasis, while four patients had stage IIIC2r disease with para-aortic lymph node involvement. Staging was determined using pelvic examination and imaging modalities, including MRI, CT, and PET-CT. FIGO: International Federation of Gynecology and Obstetrics; TC: Paclitaxel and carboplatin; BEV: Bevacizumab; PET-CT: Positron emission tomography-computed tomography

Regarding the eligibility criteria for NAC with bevacizumab, all patients had tumors measuring ≥4 cm in diameter at the time of initial diagnosis. Parametrial invasion was confirmed in all cases through pelvic gynecological examination and MRI, corresponding to T2b classification. Additionally, based on imaging assessments of retroperitoneal lymph node involvement, two patients (33.3%) were diagnosed with FIGO stage IIIC1r, while four patients (66.7%) had FIGO stage IIIC2r disease (Figure 2).

Clinical courses and treatment responses after NAC

In this study, patients received three or four cycles of a platinum-based NAC regimen in combination with bevacizumab prior to undergoing radical surgery (Figure 1). All patients completed the planned NAC cycles, followed by radical hysterectomy and retroperitoneal lymphadenectomy. The median interval from the initiation of NAC to radical surgery was 13 weeks (range: 10 to 13 weeks).

**Figure 3:**
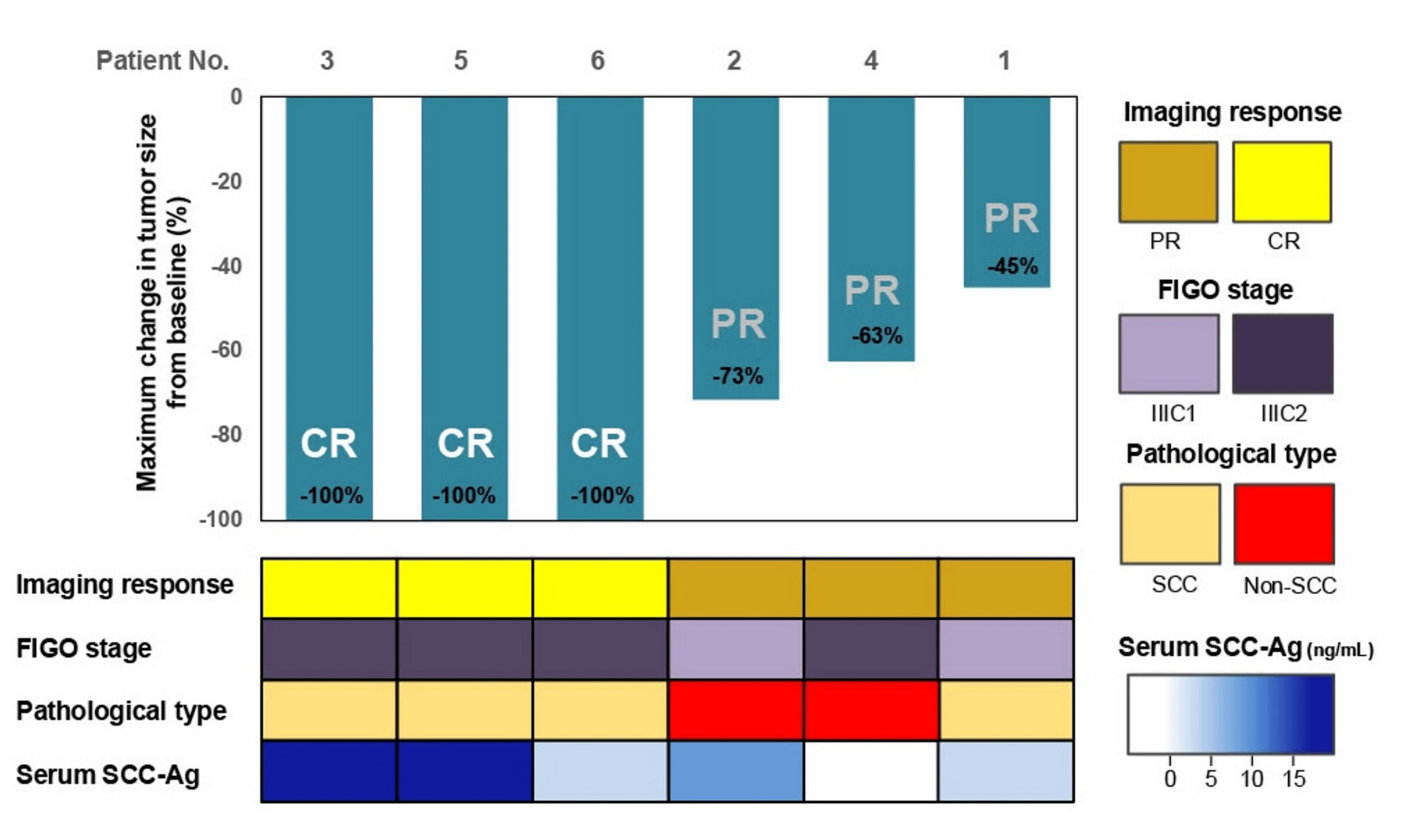
Waterfall plot illustrating the percentage change in tumor size (longest diameter) before and after NAC with bevacizumab All six patients showed substantial tumor shrinkage following NAC. Pathological complete response was achieved in three patients (50.0%), with no residual tumor observed in the surgical specimens. Corresponding treatment response, FIGO stage, histological type, and serum SCC-Ag levels are also indicated for each patient. NAC: Neoadjuvant chemotherapy; FIGO: International Federation of Gynecology and Obstetrics; SCC-Ag: Squamous cell carcinoma antigen; PR: Partial response; CR: Complete response

With respect to the efficacy of NAC, a significant reduction in tumor size was observed in all cases. Notably, pathological examination of the resected specimens revealed a pathological complete response (CR) in three of six patients (50.0%). The changes in tumor size for each case are showcased above in Figure 1 and below in Figure 3.

In the context of adjuvant therapy following radical surgery, per the pathological diagnosis of postoperative specimens, four patients with squamous cell carcinoma (66.7%) received adjuvant chemotherapy alone, whereas two patients (patients no. 2 and no. 4) with non-squamous carcinoma (33.3%) received CCRT followed by adjuvant chemotherapy (Figure 1).

Oncological survival outcomes

**Figure 4:**
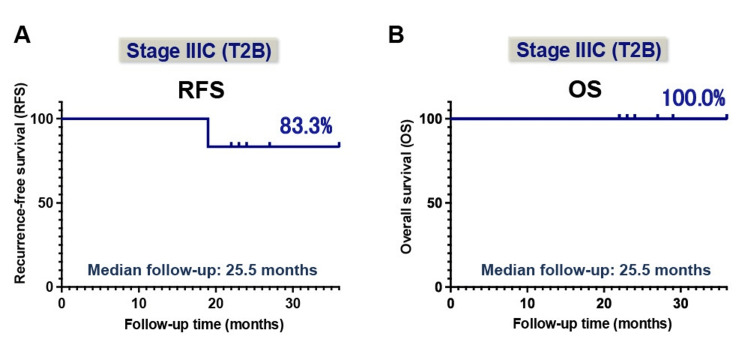
Recurrence-free survival (A) and overall survival (B) after NAC with bevacizumab followed by radical hysterectomy in six patients with FIGO stage IIIC cervical cancer At a median follow-up of 25.5 months, five patients (83.3%) remained recurrence-free. No deaths occurred during the observation period, indicating encouraging short-term oncological outcomes. NAC: Neoadjuvant chemotherapy; RFS: Recurrence-free survival; OS: Overall survival

At a median follow-up of 25.5 months (range: 22 to 36 months), five patients (83.3%) remained recurrence-free, while one patient (patient no. 5) experienced disease recurrence at 19 months after the initial treatment (Figure 4A). In the recurrent case, a solitary recurrent lesion in the left pelvic lymph node was completely resected, followed by postoperative CCRT. As a result, the patient has remained disease-free for over nine months since the surgical resection. Importantly, no deaths were reported during the study period (Figure 4B). These findings suggest that all patients achieved remarkably favorable survival outcomes, despite being diagnosed at an advanced stage (FIGO stage IIIC1/2r, T2b).

Perioperative outcomes and adverse events

The median operative time was 515 minutes (range: 485 to 610 minutes), and the median intraoperative blood loss was 473 mL (range: 345 mL to 620 mL). No patients required intraoperative blood transfusion, and no intraoperative complications or conversions to alternative procedures were observed. Regarding surgery-related adverse events, one patient developed a pelvic lymphocyst, which resolved within a short period with conservative management. Importantly, due to the use of nerve-sparing surgical techniques, none of the six patients experienced postoperative urinary dysfunction (Table 1).

**Table 1:** Operative details and perioperative complications following radical hysterectomy

Parameters	Total (n = 6)
Operative finding	
Median operative time (range in minutes )	515 (485–610)
Median blood loss (range in mL)	473 (345–620)
Blood transfusion, n (%)	0 (0.0)
Surgery-related adverse event	
Postoperative bleeding, n (%)	0 (0.0)
Ileus/Bowel obstruction, n (%)	0 (0.0)
Deep venous thrombosis, n (%)	0 (0.0)
Pelvic infection, n (%)	0 (0.0)
Urinary incontinence, n (%)	0 (0.0)
Urinary retention, n (%)	0 (0.0)
Lymphocyst, n (%)	1 (16.7)
Lymphedema, n (%)	0 (0.0)

Concerning adverse events related to NAC with bevacizumab, treatment was generally well tolerated, with most toxicities falling within acceptable clinical limits. Notably, grade 3 neutropenia was observed in five of six patients (83.3%), but no cases of febrile neutropenia were reported. In addition, hypertension attributable to bevacizumab occurred in two patients (33.3%); however, it resolved promptly with short-term antihypertensive therapy. No other severe bevacizumab-associated toxicities, including delayed wound healing, proteinuria, or gastrointestinal perforation, were observed (Table 2). Taken together, these findings indicate that NAC with bevacizumab followed by radical surgery demonstrated highly favorable perioperative safety and tolerability.

**Table 2:** Adverse events in patients receiving NAC with bevacizumab NAC: Neoadjuvant chemotherapy

Adverse events	Total n = 6	
	All grades, n (%)	Grade ≧3, n (%)
Cytotoxic chemotherapy-related adverse event		
Fatigue	3 (50.0)	0 (0.0)
Nausea	2 (33.3)	0 (0.0)
Neurotoxicity	2 (33.3)	1 (16.7)
Alopecia	6 (100)	0 (0.0)
Anemia	0 (0.0)	0 (0.0)
Leukopenia	5 (83.3)	4 (66.7)
Neutropenia	5 (83.3)	4 (66.7)
Febrile neutropenia	0 (0.0)	0 (0.0)
Thrombocytopenia	0 (0.0)	0 (0.0)
Bevacizumab-related adverse event		
Hypertension	2 (33.3)	0 (0.0)
Proteinuria	0 (0.0)	0 (0.0)
Thromboembolism	0 (0.0)	0 (0.0)
Wound dehiscence	0 (0.0)	0 (0.0)
Vesicovaginal fistula	0 (0.0)	0 (0.0)
Rectovaginal fistula	0 (0.0)	0 (0.0)
Gastrointestinal perforation	0 (0.0)	0 (0.0)

## Discussion

In this retrospective study, we evaluated the oncological efficacy and perioperative safety of NAC with bevacizumab prior to radical hysterectomy in patients with stage IIIC cervical cancer. Our findings demonstrate that this treatment strategy is feasible, well tolerated, and may offer favorable oncological outcomes, even in these high-risk cervical cancer patients with retroperitoneal lymph node metastasis.

Previous studies of NAC without bevacizumab have reported variable response rates and survival outcomes in patients with bulky or lymph node-positive cervical cancer, without consistent evidence of survival advantage over standard CCRT [8,10-12]. However, a variety of large-scale clinical trials have consistently reported five-year survival rates of approximately 65% to 75% with CCRT for locally advanced cervical cancer, indicating that there remains considerable room for therapeutic improvement [6,9,10,15,17,25,26].

In recent years, the addition of bevacizumab to platinum-based chemotherapy has demonstrated survival benefits in patients with recurrent or metastatic cervical cancer, as evidenced by the landmark Gynecologic Oncology Group (GOG) 240 trial [19]. However, although several initial studies have suggested the potential efficacy of bevacizumab-containing NAC regimens, its survival benefit and clinical role in the neoadjuvant setting for locally advanced cervical cancer remain insufficiently investigated [16,18,27].

In the present study, NAC with bevacizumab induced substantial tumor regression in all six patients. It should be noted that our pathological complete response (CR) rate of 50% exceeds those reported in previous studies [7,8,10], despite the inclusion of node-positive, high-risk patients, highlighting the potential efficacy of our treatment strategy. Our data suggest that the incorporation of bevacizumab may enhance tumor response, particularly in chemotherapy and radiation-naïve cervical cancer patients, possibly through synergistic effects, such as increased chemosensitivity mediated by anti-angiogenic mechanisms and modulation of the tumor immune microenvironment (TME), which may further promote antitumor immunity [19,28,29].

Concerning survival outcomes associated with NAC plus bevacizumab treatment, our study contributes to the limited body of evidence by demonstrating promising short-term survival: five of six patients (83.3%) remained recurrence-free at a median follow-up of 25.5 months. Given that previous large-scale trials demonstrated that most disease progressions occur within the first two years in patients with locally advanced cervical cancer treated with either NAC followed by radical surgery or CCRT [8-10], these results are particularly notable. Especially as all patients had tumors ≥4 cm with confirmed parametrial invasion (T2b), and the majority (66.7%) with para-aortic lymph node involvement, corresponding to stage IIIC2r. These findings suggest that NAC with bevacizumab followed by radical surgery may be considered a viable therapeutic option, particularly in institutions where surgical outcomes are superior to those achieved with CCRT.

From a surgical perspective, all patients successfully underwent radical hysterectomy without intraoperative complications or the need to convert to alternative procedures. Although a relatively long median operative time of 515 minutes was observed, this may be attributed to marked adhesions and extensive fibrosis around the regressed cervical tumor-likely induced by NAC, as well as the requirement for para-aortic lymphadenectomy in four of the six patients. Nevertheless, intraoperative blood loss remained within a manageable range, and none of the patients required blood transfusion. More importantly, the nerve-sparing approach resulted in preserved postoperative urinary function in all six patients. Furthermore, no serious postoperative complications were observed, providing strong support for the perioperative safety of our therapeutic strategy.

In this study, adverse events associated with NAC were manageable. Although grade 3 neutropenia was observed in 83.3% of patients, no cases of febrile neutropenia occurred. Notably, no clinically significant severe toxicities related to bevacizumab were observed. These findings support the perioperative safety of bevacizumab in carefully selected patients, consistent with previous reports suggesting that bevacizumab can be used safely in surgical settings when discontinued at an appropriate interval prior to surgery [21,30,24].

This study has several limitations. First, the small sample size limits the generalizability of the findings. Second, the retrospective design may have introduced selection bias. Third, although the follow-up duration was sufficient to detect early recurrence, long-term oncological outcomes remain undetermined. Despite these limitations, the consistency of the findings and the absence of significant complications highlight the potential value of this approach.

## Conclusions

Our findings demonstrate that NAC with bevacizumab followed by radical hysterectomy is a feasible and potentially effective treatment strategy for selected high-risk patients with locally advanced cervical cancer accompanied by lymph node metastasis in a real-world setting. This innovative multimodal approach provided high response rates, excellent perioperative tolerability, and favorable oncological outcomes. Although these initial findings are promising, additional multicenter prospective studies are urgently warranted to confirm the efficacy and safety of this therapeutic strategy to precisely delineate the subset of patients most likely to derive meaningful benefit and to further elucidate the therapeutic value of anti-angiogenic agents in the neoadjuvant setting for cervical cancer.
